# A Comparison of Midwives’ Perceptions of the Labour Care Guide and the Traditional Partograph: A Multicenter Cross-Sectional Study

**DOI:** 10.3390/healthcare14142147

**Published:** 2026-07-16

**Authors:** Sibel İçke, Beril Nisa Yaşar

**Affiliations:** Midwifery Department, Faculty of Health Sciences, Mardin Artuklu University, Mardin 47200, Türkiye; yasarberiln@artuklu.edu.tr

**Keywords:** midwifery, partograph, Labour Care Guide, intrapartum care, clinical guidelines

## Abstract

**Highlights:**

**What are the main findings?**

- The vast majority of midwives prefer the traditional partograph over the Labour Care Guide (LCG) as a more suitable and efficient tool due to long-standing usage habits and ease of application.
- The LCG is perceived as a tool that increases the workload due to the abundance of parameters it contains and is considered overly detailed for clinical practice.

**What are the implications of the main findings?**

- It has been observed that midwives with high professional competence, job satisfaction, and clinical experience are more inclined to adopt new monitoring methods and adapt to the implementation processes.
- For the new guidelines to gain acceptance in the field, it is critically important to go beyond theoretical training and plan for workload adjustments, staff competency, and practice-oriented in-service training.

**Abstract:**

**Background/Objectives**: While the traditional partograph remains widely used in labor monitoring, concerns about its clinical effectiveness have led to the introduction of the World Health Organization’s Labour Care Guide (LCG) in 2020, raising questions about its acceptance in practice. This study aimed to determine midwives’ perceptions of these two tools, their usage preferences, and the influencing factors. **Methods**: This multicenter cross-sectional study was conducted with 153 midwives working in public and university hospitals in Mardin, Batman, and Hakkari between 7 April and 30 June 2025. Data were collected using a socio-demographic form and analyzed with descriptive statistics, Chi-square tests, and logistic regression. **Results**: Findings showed that 89.5% of midwives considered the partograph more appropriate, and 85.6% found it more practical. In contrast, 62.1% rated the LCG as poor, and 77.8% reported that not all its parameters were necessary. Midwives with high job satisfaction used the partograph significantly more actively. Those working in public hospitals were 2.83 times more likely to find the LCG useful than those in university hospitals, while midwives with higher perceived professional competence were 2.29 times more likely to favor the LCG. **Conclusions**: Midwives perceive the traditional partograph as more efficient, whereas the LCG is viewed as overly detailed and increasing workload. The findings suggest that beyond theoretical training, successful LCG implementation requires workload regulation, adequate staffing, and practice-oriented in-service training.

## 1. Introduction

Effective and safe monitoring of labour is a fundamental component in protecting maternal and neonatal health. Monitoring tools used during the intrapartum period enable objective assessment of the labour process, help in the early detection of complications, and play a significant role in preventing unnecessary obstetric interventions [1]. In this regard, the partograph, having been widely used in clinical practice for many years, has been one of the primary tools aimed at monitoring the progress of labour within a standardized framework [2].

The primary objective of the partograph is to detect prolonged labour at an early stage, prevent obstetric complications, and reduce maternal and perinatal morbidity [3]. However, recent studies have referred to various limitations concerning its clinical effectiveness and applicability. The literature highlights issues such as incomplete or incorrect completion of the partograph, its inconsistent use due to heavy workload, and its limited impact on labour outcomes [4]. Furthermore, the traditional approach, based on the assumption that cervical dilatation progresses at a constant rate, does not adequately reflect the individual and dynamic nature of the labour process [5].

In line with these criticisms, the World Health Organization (WHO) emphasized in its 2018 recommendations that labour should be managed using a woman-centred, individualized, and holistic approach rather than relying solely on cervical dilatation patterns [1]. Building on these recommendations, the Labour Care Guide (LCG) was introduced by the WHO in 2020 as a next-generation intrapartum monitoring tool and an alternative to the traditional partograph for routine labour monitoring [6,7]. Unlike the traditional partograph, which primarily focuses on monitoring cervical dilatation and labour progress, the LCG adopts a more comprehensive, woman-centred approach. It incorporates assessment of maternal well-being (e.g., vital signs, pain, and supportive care), fetal condition, labour progress, and clinical decision-making, while emphasizing respectful maternity care and individualized labour management rather than adherence to a fixed rate of cervical dilatation. The LCG therefore aims not only to facilitate timely identification of abnormal labour but also to improve the quality, safety, and experience of intrapartum care by supporting evidence-based, individualized decision-making and respectful maternity care [6,7].

However, the development of new clinical guidelines within health systems does not necessarily ensure their effective implementation in practice. The adoption of innovations in clinical practice depends not only on the strength of scientific evidence but also on healthcare professionals’ perceptions of the innovation, their evaluations of its ease of use, and their levels of professional self-efficacy. The primary barriers encountered in the implementation of clinical guidelines include workload, time constraints, lack of organizational support, and the complexity of the guidelines [4,8,9]. In particular, in high-intensity clinical settings such as delivery rooms, the adoption of a new monitoring tool represents not only a technical change but also a process of behavioral and organizational transformation. Healthcare professionals’ perceptions and attitudes toward new practices play a determining role in this process. The literature indicates that professional acceptance and perceived ease of use are critical factors in the adoption of clinical practice guidelines [8,9].

As the primary practitioners of the labour process, midwives play a key role in translating intrapartum care models into clinical practice. Therefore, comparing professional perceptions of the traditional partograph and the LCG, and identifying the factors influencing these perceptions, constitutes an essential requirement in terms of both clinical practice and health policy. Despite the growing global interest in the LCG, evidence comparing healthcare professionals’ perceptions of the LCG and the traditional partograph, particularly in clinical settings with low- and middle-level resources, remains limited [6,10]. It is essential that midwives understand the level of acceptance of these tools and their perceived ease of use for successful application.

Therefore, this study aims to comparatively examine midwives’ perceptions of the traditional partograph and the LCG, their usage preferences, and the socio-demographic and professional factors affecting these preferences.

## 2. Materials and Methods

### 2.1. Type of Research

This multicenter, cross-sectional study aims to compare midwives’ perceptions and preferences regarding the traditional partograph and the LCG model.

### 2.2. Setting and Duration

This multicenter study was conducted in Mardin, Batman, and Hakkari, three provinces in eastern and southeastern Türkiye. The study period covered 7 April 2025 to 30 June 2025. Although these provinces differ in healthcare delivery, patient volume, and working conditions, they serve as active centers for maternity services. In this respect, the study may reflect the diverse experiences of midwives working in various clinical contexts.

### 2.3. Population and Research Sample

The study population consisted of 187 midwives actively working in delivery rooms across the three relevant provinces (Mardin: 105, Batman: 56, Hakkari: 26). An a priori power analysis was conducted using G*Power 3.1.9.7 to determine the minimum required sample size before data collection. Assuming a medium effect size (*d* = 0.5), which is in line with Cohen’s conventional benchmark for behavioral and attitudinal research and was considered a conservative planning value for a cross-sectional survey of midwives’ perceptions and preferences, a sample size of 124 participants was required to achieve 80% statistical power at a 0.05 significance level. Rather than applying a probabilistic sampling method, the study aimed to include the entire accessible population across the three provinces. It was completed with 153 midwives who participated voluntarily (response rate: 81.8%). This high participation rate increases the representativeness of the findings by reducing selection bias and supports the external validity of the observational design.

### 2.4. Data Collection Tools

Data collection was conducted using a “Sociodemographic and Professional Characteristics Form” developed by the researchers in line with the literature [11,12]. The form included items on age group, place of residence, marital status, educational level, institution of employment, years of delivery room experience, whether the participant had chosen to work in the delivery room voluntarily, job satisfaction, intention to leave the delivery room, previous partograph training, satisfaction with training, active use of the partograph, perceived competence in partograph use, opinions regarding the WHO-recommended LCG, and comparative evaluations of the traditional partograph and the LCG. The questionnaire also assessed whether all parameters in the new partograph were considered necessary and whether the partograph was deemed necessary for labour monitoring. Response options were primarily categorical and included Yes/No, Yes/No/Undecided, Good/Poor/No opinion, Old/New, and Necessary/Sometimes Necessary/Not necessary. Prior to the main study, the questionnaire was piloted on a small group of midwives to evaluate clarity, readability, and feasibility, with minor wording changes made based on the pilot feedback.

### 2.5. Data Collection

Data were collected using a researcher-developed questionnaire. After the participants were informed about the study and provided written informed consent, they were invited to complete the forms during the training sessions organized by the Provincial Health Directorates. The questionnaires were administered under the supervision of the researchers.

Inclusion Criteria:

- Volunteering to participate in the study
- Practicing midwifery actively
- Working in the delivery room

Exclusion Criteria:

- Refusal to participate in the study

### 2.6. Ethical Consideration

Approval for the research was obtained from the Mardin Artuklu University Non-Interventional Ethics Committee (Date and Ref. No.: 01/05/2025-193486). The study was conducted in accordance with the principles of the Declaration of Helsinki, receiving written informed consent from all participants.

### 2.7. Data Analysis

The data obtained from the study were analyzed using IBM SPSS Statistics version 22 (IBM Corp., Armonk, NY, USA) software. The participants’ descriptive characteristics and views on partograph use were evaluated using frequencies and percentages, while the age variable was analyzed using mean and standard deviation. Categorical variables were analyzed using the chi-square test. To examine how independent variables predicted the preference for either the partograph or the LCG, logistic regression analysis was performed. Model fit was assessed using the Omnibus test of model coefficients, Nagelkerke *R*^2^, and the Hosmer–Lemeshow test. The statistical significance level was set at *p* < 0.05.

## 3. Results

The midwives’ sociodemographic and professional descriptive characteristics are presented in Table 1. The mean age of the participating midwives (n = 153) was 29.56 ± 5.54 years (Min: 22, Max: 48), with 66% (n = 101) aged 30 years or younger. Of them, 62.1% were single and 96.0% held a bachelor’s degree, with 66% being employed in state hospitals and 34% in university hospitals. Professional experience in the delivery room varied, with 35.3% having 0–2 years of experience, while 32.0% had more than 5 years. The proportion of midwives working in the delivery room by choice was 78.4%. While the satisfaction rate with working in this unit was 59.5%, 20.9% of the midwives expressed a desire to leave the unit. An analysis of the reasons for desiring to leave identified excessive workload as the primary factor (87.5%), followed by health problems (6.3%), work environment (3.1%), and issues related to perceived injustice (3.1%).

The midwives’ views and preferences regarding the partograph and the LCG are presented in Table 2. A vast majority of them (91.5%) had previously received training on the partograph and 88.6% of those trained expressed satisfaction with the training. Additionally, 85.0% of the midwives reported using the partograph actively, and 83.0% considered themselves sufficient and competent in its use.

Considering perceptions toward the LCG, 62.1% of the midwives described the guide as “poor,” while only 20.9% found it favorable. When comparing the traditional partograph with the new LCG, 89.5% of the midwives evaluated the partograph as more appropriate, 73.2% as more efficient, and 85.6% as a more useful tool.

Regarding the necessity of all parameters within the LCG, 77.8% of the midwives responded “no,” indicating that they found the current version of the guide too detailed. Regarding the general necessity of a partograph as a tool for labour monitoring, 66.0% of the midwives argued that using a partograph is “always necessary,” while 22.2% stated it might sometimes be necessary, and 11.8% stated it is not necessary.

These findings suggest that familiarity with the traditional partograph and the perceived ease of use of this form may play a significant role in determining midwives’ preferences.

Table 3 presents the distribution of the midwives’ views regarding the partograph and the LCG according to their descriptive and professional characteristics. The findings show no statistically significant difference between the midwives’ age groups and their active use of the partograph, their evaluation of the LCG, or which tool they found more appropriate, efficient, or useful (*p* > 0.05).

Similarly, there was no significant correlation between the type of institution where the midwives worked and their active use of the partograph, or their perception of the new guide (*p* > 0.05). While the preference between the partograph and LCG did not change by years of delivery room experience, years of experience significantly influenced whether midwives felt professionally “sufficient and competent” (*p* = 0.023). One of the significant and prominent findings is the correlation between job satisfaction and partograph use. The rate of active partograph use among those satisfied with their job was much higher than among those neither satisfied nor dissatisfied (*p* = 0.000). In addition, having previously received partograph training had a significant impact on whether the LCG was perceived as “good” or “poor” (*p* = 0.033).

The logistic regression analysis examined the demographic and professional factors affecting the likelihood of midwives finding the LCG “more useful” (Table 4). The non-significant result of the Hosmer and Lemeshow test (*p* = 0.748) indicates that the model’s fit with the data is good and there is no significant difference between observed and predicted values.

Nagelkerke *R*^2^ value is 0.102, which indicates that the independent variables in the model explain approximately 10.2% of the variation in “utility preference.” In social sciences and health research, this rate is considered a reasonable baseline for behavioral preferences.

There was no significant correlation between the institution variable and the perceived utility of the LCG (*p* = 0.103). However, a non-significant trend was observed, indicating that those working in state hospitals were more likely to find the guide useful (Exp(B) = 2.831).

There was no significant correlation between feeling professionally sufficient/competent and deeming the LCG useful (*p* = 0.350). However, there was a non-significant trend indicating that these healthcare professionals were more likely to find the guide useful (Exp(B) = 2.831).

Midwives with 0–2 years and 3–5 years of delivery room experience were less likely to find the LCG useful compared to those with 6 or more years of experience (0.49 and 0.56, respectively).

## 4. Discussion

The findings of this study indicate that the vast majority of midwives perceived the traditional partograph as more appropriate, efficient, and useful than the newly introduced LCG. In addition, a significant portion of participants described the LCG as a complex and detailed tool that may increase workload. These findings suggest that professional perceptions may influence preferences regarding intrapartum monitoring tools. In the present study, most midwives preferred the traditional partograph and perceived the LCG as more complex. However, because perceptions of implementation processes were not directly assessed, these findings should be interpreted cautiously. These findings are consistent with previous studies suggesting that healthcare professionals tend to prefer familiar and less complex tools, even when newer alternatives offer theoretical advantages.

### 4.1. Preference for the Traditional Partograph

The preference of the majority of midwives for the traditional partograph may reflect their greater familiarity with this tool in routine clinical practice. Previous studies have suggested that long-standing clinical tools are often more readily accepted because healthcare professionals are more familiar with their use and integration into clinical workflows [3,4,13]. However, the present study did not directly assess the reasons underlying these preferences [4]. This discrepancy suggests that healthcare professionals’ preferences may be shaped more by factors such as ease of use and procedural familiarity rather than solely by evidence-based efficacy.

### 4.2. Negative Perception Toward the LCG

A considerable proportion of midwives evaluated the LCG negatively. This finding is consistent with previous studies reporting that newly introduced clinical guidelines may initially be perceived as more complex or demanding in routine practice [8,9,13]. The LCG, introduced by the WHO in 2020, offers a comprehensive model based on woman-centred care and individualized labour monitoring [6,14]. Compared with the traditional partograph, the LCG requires documentation of a broader range of maternal, fetal, and clinical care parameters. This broader scope may contribute to perceptions that the guide is more detailed and time-consuming in routine clinical practice.

Previous studies have identified workload, time constraints, guideline complexity, and lack of organizational support as important barriers to the implementation of clinical guidelines [8,9,13]. In addition, Laisser et al. (2021) reported usability concerns regarding the LCG, including small font size and limited space for documentation [15]. Similarly, in the present study, most midwives perceived the LCG as overly detailed and not all of its parameters were necessary. These findings are consistent with previous reports suggesting that usability and perceived complexity may influence healthcare professionals’ acceptance of new clinical tools.

### 4.3. Adoption of Innovations and Behavioral Factors

Although the present study did not directly assess behavioral or cognitive determinants of guideline adoption, previous research suggests that healthcare professionals’ perceptions of usefulness and ease of use may influence their willingness to adopt new clinical practices [8,9]. Our findings, showing a preference for the traditional partograph over the LCG, are consistent with the current literature. Previous studies have suggested that healthcare professionals’ attitudes toward new practices may influence their integration into clinical routine. Further, they have identified perceived ease of use and usefulness as important predictors of behavioral intention in the adoption of clinical guidelines [9].

In the present study, many midwives perceived the LCG as more complex and detailed than the traditional partograph. Although this study did not directly evaluate implementation outcomes, this finding is consistent with previous studies suggesting that perceived complexity and workload may influence the acceptance of new clinical guidelines [8,9,13].

### 4.4. Professional Experience and Self-Efficacy

The present study found a significant correlation between delivery room experience and perceived professional competence. This finding is consistent with Bandura’s self-efficacy theory, which proposes that greater experience may contribute to stronger confidence in performing professional tasks [16]. However, because self-efficacy was not directly measured, this interpretation should be considered theoretical rather than evidence derived from the present study. In this context, midwives with higher professional experience and perceptions of self-efficacy could be more open to new clinical tools [8].

However, the lack of statistically significant predictors in the logistic regression analysis suggests that preferences regarding the LCG may not be fully explained by the demographic and professional variables included in the present model. These findings suggest that factors other than the demographic and professional variables included in the present model may contribute to preferences for the LCG. Future studies should further explore organizational and system-level factors influencing guideline adoption.

### 4.5. Education and the Adoption of Clinical Guidelines

This study found a significant correlation between receiving partograph training and the evaluation of the LCG. This finding suggests that educational processes may play an important role in shaping healthcare professionals’ attitudes and perceptions toward clinical tools. The literature reports that the level and quality of training are crucial factors influencing healthcare professionals’ adoption of new clinical practices [8,9].

The fact that the partograph has been a part of training programs for many years may have contributed to midwives developing a higher level of familiarity and confidence in this tool. In contrast, the relatively new implementation of the LCG and its limited integration into training processes may partly explain the negative perception toward the guide. The literature further emphasizes that a lack of training is a major barrier to the implementation of new clinical guidelines [9].

These findings suggest that merely introducing a guide is not enough for the integration of new intrapartum monitoring tools into clinical practice; yet structured and practice-based training programs must also be organized.

### 4.6. Job Satisfaction and Clinical Practice

This study identified a significant correlation between job satisfaction and active use of the partograph. Although organizational factors were not directly evaluated, previous studies have reported that work environment and job satisfaction may influence adherence to clinical protocols [17]. Together with previous evidence, these findings suggest that the work environment may influence the use of intrapartum monitoring tools.

### 4.7. Clinical and Political Implications

The present findings suggest that theoretical training alone may not be sufficient for the successful implementation of new intrapartum monitoring guides. The present findings suggest that successful implementation of the LCG may require not only educational initiatives but also consideration of workload and clinical workflow. Although the correlation between institution type and perceived usefulness of the LCG was not statistically significant, the observed trend may reflect differences in institutional practices or organizational contexts. Future implementation studies are needed to determine whether institutional factors influence the acceptance and implementation of the LCG. Future implementation studies are also needed to determine the most effective implementation strategies.

In addition, systematically evaluating feedback from healthcare professionals and adapting the guide to field conditions may facilitate the adoption process. The literature also emphasizes that organizational support and contextual adaptation are of critical importance in the implementation of clinical guidelines [8].

### 4.8. Strengths and Limitations

This study is significant because it is one of the few studies comparing midwives’ perceptions and preferences regarding the traditional partograph and the LCG model. Its multicenter design across three distinct provinces is a major strength, as it reflects diverse clinical environments and working conditions. Although these provinces are located within the same geographical region, variations in healthcare delivery, workload, and institutional structures enhance the transferability of the findings to similar contexts. Furthermore, reaching a large majority of the target population (81.81% participation rate) reduces selection bias and strengthens external validity. The study included only midwives actively working in the delivery room and responsible for completing both tools.

The use of multivariate analysis to account for potential confounding factors strengthens the analytical depth of the results. Being conducted during the transition period of the new intrapartum monitoring model into field application is another strength of the study, providing updated and practice-oriented data on how health policy changes are perceived at the clinical level.

The study also has certain limitations. First, due to the cross-sectional design, the findings do not allow for causal inferences but rather indicate associations between variables. Second, the relatively low number of participants who found the LCG “more useful” (n = 22) may have resulted in wide confidence intervals for some estimates in the multivariate logistic regression analysis, so these results should be interpreted with caution. Third, important contextual variables such as staffing ratios, patient load, previous exposure to the LCG, and resource availability were not included in the model, which may have limited its explanatory power. Finally, the exclusion of midwives working in private hospitals limits the assessment of different institutional dynamics and may reduce the transferability of the findings to other care settings.

## 5. Conclusions

Midwives in this study generally preferred the traditional partograph over the LCG, considering it more appropriate, efficient, and useful. The LCG was perceived as overly detailed and more likely to increase workload. These findings suggest that the successful implementation of new intrapartum monitoring tools depends not only on evidence-based recommendations but also on usability, workflow compatibility, and working conditions.

## Figures and Tables

**Table 1:** Sociodemographic and Professional Descriptive Characteristics of Midwives.

Characteristics	N	%
**Age group**		
30 years or younger	101	66.0
Over 30 years	52	34.0
**Educational status**		
Associate degree	3	2.0
Bachelor’s degree	147	96.0
Graduate degree	3	2.0
**City of participation**		
Mardin	81	52.9
Batman	51	33.3
Hakkari	21	13.8
**Institution of employment**		
State hospital	101	66.0
University/Training and Research Hospital (EAH)	52	34.0
**Delivery room experience (years)**		
0–2 years	54	35.3
3–5 years	50	32.7
More than 5 years	49	32.0
**Working in the delivery room by choice**		
Yes	120	78.4
No	33	21.6
**Satisfaction with working in the delivery room**		
Satisfied	91	59.5
Not satisfied	24	15.7
Neither satisfied nor dissatisfied	38	24.8
**Desire to leave the delivery room**		
Yes	32	20.9
No	121	79.1
**Total**	**153**	**100**

N: Number, %: Percentage.

**Table 2:** Midwives’ Opinions and Preferences Regarding Partograph and LCG.

Characteristics	N	%
**Previous partograph training status**		
Received	140	91.5
Did not received	13	8.5
**Satisfaction with training received**		
Yes	124	88.6
No	16	11.4
**Active use of partograph**		
Yes	130	85.0
No	23	15.0
**Perceiving themselves sufficient and competent**		
Yes	127	83.0
No	26	17.0
**Opinion on LCG**		
Good	32	20.9
Poor	95	62.1
No opinion	26	17.0
**The more appropriate method**		
Partograph	137	89.5
LCG	16	10.5
**The more efficient method**		
Partograph	112	73.2
LCG	41	26.8
**The more useful method**		
Partograph	131	85.6
LCG	22	14.4
**Necessity of all parameters in LCG**		
Necessary	34	22.2
Not necessary	119	77.8
**Necessity of partograph in labour monitoring**		
Not necessary	18	11.8
Sometimes necessary	34	22.2
Always necessary	101	66.0
**Total**	**153**	**100**

N: Number, %: Percentage. LCG: Labour Care Guide.

**Table 3:** Distribution of Midwives’ Opinions on Partograph and LCG According to Descriptive and Professional Characteristics.

**Characteristics**	**Opinions on the Partograph and LCG**					**Total**
**Age Group **	**Active Use of Partograph**					
	**Yes**		**No**			
30 years or younger	84		17			101
Over 30 years	46		6			52
*Statistical value (X* ^2^ *, Fisher’s), p-value*	*X*^2^ = 0.753 *p* = 0.386					
**Age group**	**Opinion on LCG**					
	**Good**		**Poor**		**No opinion**	
30 years or younger	18		64		19	101
Over 30 years	14		31		7	52
*Statistical value (X* ^2^ *, Fisher’s), p-value*	*X*^2^ = 2.016 *p* = 0.365					
**Age group**	**The more appropriate method**					
	**Partograph**		**LCG**			
30 years or younger	91		10			101
Over 30 years	46		6			52
*Statistical value (X* ^2^ *, Fisher’s), p-value*	*X*^2^ = 0.098 *p* = 0.754					
**Age group**	**The more efficient method**					
	**Partograph**		**LCG**			
30 years or younger	78		23			101
Over 30 years	34		18			52
*Statistical value (X* ^2^ *, Fisher’s), p-value*	*X*^2^ = 2.454 *p* = 0.117					
**Age group**	**The more useful method**					
	**Partograph**		**LCG**			
30 years or younger	89		12			101
Over 30 years	42		10			52
*Statistical value (X* ^2^ *, Fisher’s), p-value*	*X*^2^ = 1.50 *p* = 0.220					
**Delivery room experience (years)**	**The more appropriate method**					
	**Partograph**		**LCG**			
0–2 years	50		4			54
3–5 years	45		5			50
More than 5 years	42		7			49
*Statistical value (X* ^2^ *, Fisher’s), p-value*	*X*^2^ = 1.315 *p* = 0.518					
**Delivery room experience (years)**	**Perceiving themselves sufficient and competent**					
	**Yes**		**No**			
0–2 years	39		15			54
3–5 years	46		4			50
More than 5 years	42		7			49
*Statistical value (X* ^2^ *, Fisher’s), p-value*	*X*^2^ = 7.574 ***p* = 0.023**					
**Institution of employment**	**Opinion on LCG**					
	**Good**		**Poor**		**No opinion**	
State hospital	22		59		20	101
University/Training and Research Hospital (EAH)	10		36		6	52
*Statistical value (X* ^2^ *, Fisher’s), p-value*	*X*^2^ = 2.133 *p* = 0.344					
**Institution of employment**	**Active use of partograph**					
	**Yes**		**No**			
State hospital	86		15			101
University/Training and Research Hospital (EAH)	44		8			52
*Statistical value (X* ^2^ *, Fisher’s), p-value*	*X*^2^ = 0.008 *p* = 0.930					
**Satisfaction with working in the delivery room**	**Active use of partograph**					
	**Yes**		**No**			
Satisfied	85		6			91
Not satisfied	21		3			24
Neither satisfied nor dissatisfied	24		14			38
*Statistical value (X* ^2^ *, Fisher’s), p-value*	*X*^2^ = 19.346 ***p* = 0.000**					
**Partograph training status**	**Opinion on LCG**					
	**Good**	**Poor**		**No opinion**		
Received	32	87		21		140
Did not received	0	8		5		13
*Statistical value (X* ^2^ *, Fisher’s), p-value*	*X*^2^ = 6.825 ***p* = 0.033**					
**Toplam**						**153**

*X*^2^: Chi-square, *p*: *p*-value. LCG: Labour Care Guide.

**Table 4:** Results of the Logistic Regression Analysis on the Usability of LCG.

Independent Variables	B	S.E.	Wald	df	*p*	OR (Exp B)	95% Cl (Lower–Upper)
**Training (yes)**	−0.317	0.941	0.021	1	0.884	0.872	0.138–5.507
**Age group (30 years and under)**	−0.346	0.587	0.347	1	0.556	0.707	0.224–2.236
**Educational status**			0.185	2	0.911		
− Associate degree	−0.382	1.881	0.041	1	0.839	0.683	0.017–27.227
− Bachelor’s degree	−0.590	1.425	0.171	1	0.679	0.555	0.034–9.046
**Delivery room experience (year)**			1.094	2	0.579		
− 0–2 years	−0.699	0.711	0.965	1	0.326	0.497	0.123–2.005
− 3–5 years	−0.573	0.684	0.700	1	0.403	0.564	0.147–2.157
**Institution of employment**	1.041	0.638	2.664	1	0.103	2.831	0.811–9.875
**Working by choice**	−0.290	0.636	0.208	1	0.648	0.748	0.215–2.603
**Work satisfaction**			0.647	2	0.724		
− Yes	0.431	0.646	0.445	1	0.505	1.539	0.434–5.454
− No	0.605	0.813	0.555	1	0.456	1.832	0.372–9.014
**Adequacy perception**	0.829	0.887	0.873	1	0.350	2.291	0.403–13.035
**City of participation**			1.818	2	0.403		
− Mardin	−0.189	0.83	0.058	1	0.809	0.828	0.178–3.841
− Batman	0.584	0.871	0.449	1	0.503	1.792	0.325–9.880
**Constant**	−2.194	2.337	0.881	1	0.348	0.112	

Nagelkerke *R*^2^ = 0.102; Hosmer and Lemeshow Test: *X*^2^ = 5.087, *p* = 0.748. The reference category for the dependent variable is “Partograph.” B = regression coefficient, S.E. = standard error, Wald = Wald test statistic, df = degrees of freedom, *p* = *p*-value, OR (Exp B) = odds ratio, and 95% CI (Lower–Upper) = 95% confidence interval lower and upper limits. LCG: Labour Care Guide.

## Data Availability

The data presented in this study are available on request from the corresponding author. The data are not publicly available due to privacy and ethical restrictions.

## Abbreviations

The following abbreviations are used in this manuscript:

WHO	World Health Organization
LCG	Labour Care Guide
